# The development and validation of multidimensional workplace compassion scale: Linking its conceptualization and measurement

**DOI:** 10.3389/fpsyg.2023.1142309

**Published:** 2023-03-30

**Authors:** Anshul Mandliya, Jatin Pandey

**Affiliations:** Indian Institute of Management Indore, Indore, India

**Keywords:** workplace compassion, scale development, organizational compassion, positive organizational scholarship, measuring compassion at work

## Abstract

Organizational compassion is a powerful force that possesses the capability to move individuals and groups towards a common good. Research on organizational compassion or compassion in the workplace has discussed its potential to reduce individual suffering and enhance positive emotions, wellbeing, and dignity. The existing literature lacks a valid and reliable measure of workplace compassion that follows the recent conceptualization of organizational compassion. This research presents the development and validation of workplace compassion scale. The scale development process consisted of four studies with a total sample of 947 respondents. In study 1, we developed the items for the new measure, by considering the four-factor conceptualization of organizational compassion. Study 2 and 3 focuses on item purification and testing the model fit indices of the proposed scale. In study 4, we followed a time separated design to test the nomological network and discriminant validity of the workplace compassion scale. The final workplace compassion scale consists of 12 items that measure other-oriented/expressed compassion in the workplace. The scale is beneficial for providing impetus to future quantitative research in organizational compassion.

## 1. Introduction

Compassion has been a popular human virtue for many centuries (Pommier et al., 2020). Literature on religion, evolutionary sciences, psychology, medicine, and many other fields has discussed the positive effects of compassion on individuals and society (Frost, 1999; Kanov et al., 2004; Strauss et al., 2016). Considering these positive effects of compassion, scholars in management have also studied compassion at work and its ability to bring positive outcomes at the personal, relational, and organizational levels (Dutton et al., 2006, 2014; DeCelles and Anteby, 2020; Schabram and Heng, 2021). Compassion emanates from suffering which is an inevitable part of one’s life; it (suffering) threatens their well-being in the workplace (Kanov et al., 2004).

Research demonstrates that compassion possesses the capability to not only reduce the sufferer’s pain but also enhances positive emotions in the giver, receiver, and witnesses of compassionate acts (Lilius et al., 2008; Dutton et al., 2014). Compassion in the management literature is conceptualized as an interpersonal process that involves noticing the pain, having empathetic concerns toward the sufferer, engaging in the process of sensemaking of the sufferer’s situation, and finally responding to alleviate the sufferer’s pain (Atkins and Parker, 2012; Dutton et al., 2014). These four processes are not necessarily successive (Dutton et al., 2014). Organizational compassion also captures larger team and organizational level conceptualization that covers organizing for compassion and other macro-level responses (Madden et al., 2012; Miller et al., 2012; Grimes et al., 2013); however, the focus of this paper is individual level compassionate response in the workplace.

Scales have been previously developed which measured some or other forms of compassion, such as experienced compassion scale (Lilius et al., 2008), Compassion scale (Pommier et al., 2020), Lovingkindness-Compassion Scale (Cho et al., 2018), Sussex-Oxford Compassion Scales (Gu et al., 2020), and scales which measured self-compassion (Neff, 2003; Raes et al., 2011; Neff et al., 2021); however, they lacked specific focus on workplaces which are intertwined with organizational processes, structures, roles, work goals and others. We develop workplace compassion scale specially to measure individual compassionate responses in the workplace (a more granular form of organizational compassion). In the workplace setting, job and organizational goals can overpower personal tendency to display compassion to others. Management literature emphasizes that workplaces are intertwined with personal (formal role, professional identity, job demand), relational (relational networks, access to resources, team membership, status, power), and organizational factors (shared values, norms, organizational practices, structure) which affect the process and outcomes of compassion (Dutton et al., 2006, 2014). In light of the other compassion measures’ inapplicability to capture workplace compassion and a growing interest of organizational behavior scholars and I/O (Industrial/organizational) psychologists, there is a need for a valid and reliable measure of workplace compassion.

In addition to this, the existing literature on workplace compassion has majorly tried to capture it through qualitative interviews or case-based methodologies (Dutton et al., 2006; Lilius et al., 2008, 2011; DeCelles and Anteby, 2020). Quantitative methods in management have predominantly captured the experienced compassion (Lilius et al., 2008), team compassion (Wee and Fehr, 2021), and psychological compassion climate (Nolan et al., 2022) at work but not an individual’s expressed compassion in the workplace. Thus, the article attempts to develop a measure of workplace compassion. The primary contribution of the papers is that it reports the development and validation of the workplace compassion scale, which represents the other-oriented compassion displayed by an individual in the workplace. In the present times, compassion has become a prominent area of research in management, and many scholarly works have emphasized the importance of compassion in the workplace (Dutton et al., 2006; Lilius et al., 2008; DeCelles and Anteby, 2020; Schabram and Heng, 2021). Therefore, it is important to measure workplace compassion. To accomplish the objective of developing a workplace compassion scale, we conducted four studies. These studies are designed to follow the best practices of scale development and validation for creating a psychometrically sound measure of workplace compassion (MacKenzie et al., 2011; Otaye-Ebede, 2018; Gu et al., 2020; Nolan et al., 2022). First study focuses on item generation and content validation, in line with the current conceptualization of organizational compassion (Dutton et al., 2014). Second study demonstrates the item purification process with exploratory factor analysis. Third study shows the factor structure of the four-factor workplace compassion scale. Fourth study confirms the nomological and discriminant validity of the workplace compassion scale in relation to other constructs. In this paper, we have used the terms organizational compassion (micro-level understanding), compassion at work, and workplace compassion interchangeably.

## 2. Literature review

Compassion has been a focus of research in many fields from neuroscience to psychology due to its positive physiological and psychological effects (Gilbert, 2020). In addition to its positive personal effects, it also promotes prosocial behaviors within a particular community or group (Gilbert, 2019). The early accounts of research on compassion suggest that it involves three processes-recognition (appreciation) of others’ suffering, having a sympathetic reaction to their distress, and intention to alleviate their suffering (Carr, 1999). Compassion seems to challenge the Taylorian ideals which considered employees as production resources through scientific management (George, 2014). Taylorian ideals of scientific management created a culture of ignorance and avoidance toward human emotions in the workplace (Simpson and Berti, 2020). Compassion helps break these ideals and brings humanistic values to the organization, that can garner collective actions in difficult times (Dutton et al., 2006). Compassion at an individual level involves noticing, feeling, and responding to others’ suffering (Kanov et al., 2004). While compassion at an organizational level enables the legitimization, propagation, and coordination of activities through structures, processes, practices, values, and routines to collectively notice, feel, and act toward events of suffering (Kanov et al., 2004; Dutton et al., 2006; Rynes et al., 2012).

The recent conceptualizations in compassion suggest that compassionate responding also involves a process of appraising, where the caregiver evaluates the sufferer’s condition and context before they express compassion (Atkins and Parker, 2012). This process of psychological appraisals is also theorized as sensemaking, where the individual is involved in the interpretative work of comprehending the situation of the sufferer (Dutton et al., 2014). Compassion is considered to be beneficial for individuals, organizations, and societies. The literature on compassion has accumulated a wide range of definitions to understand compassionate responding and its psychometric properties (Strauss et al., 2016). A recent literature review on compassion provided a comprehensive understanding and comparison of its definitions and measures along with their shortcomings (Strauss et al., 2016). To avoid any repetition, we have excluded these measures of compassion from our study and comparison. Also, the focus of this paper is workplace compassion, hence we have included those scales on compassion which are related to work or workplace or management or are recently developed and used in the management literature. Table 1 provides a summary of recent measures on compassion and their limitations in measuring organizational compassion. Table 1 is an extension of Strauss et al. (2016) work with organizational compassion as the focal construct of the analysis.

**Table 1:** Psychometric properties of measures of compassion and organizational compassion.

Sr. No.	Scale Name	Authors (year)	Organizational context captured?	Content validity: sub-factors (Noticing, empathizing, sensemaking, and acting) captured	Content validity: Q-sort	Proposed factor structure	Item loading and factor structure: EFA	Support for factor structure: CFA and model fit	Internal consistency: Cronbach alpha (for total scale and subscales)
1	Experienced compassion	Lilius et al. (2008)	Yes	No, it captured compassion on the job, from supervisor, and from coworker	No	Single factor	No	No	0.79
2	Compassion scale	Pommier et al. (2020)	No	No, compassion is captured from Neff’s theoretical model	No	4 factors: K = kindness; CH = common humanity; M = mindfulness; I = indifference;	Yes	Yes	0.78 to 0.90 across samples (yes for all)
3	Lovingkindness-Compassion Scale	Cho et al. (2018)	No	No, compassion captured Buddhist tradition	No	3 factors: (loving-kindness, compassion and self-centeredness)	Yes	Yes	0.85 (yes for all)
4	Sussex-Oxford Compassion Scales (SOCS)	Gu et al. (2020)	No	Yes- noticing, empathizing, acting No- sensemaking	No	5 factors: (a) recognizing suffering, (b) understanding the universality of suffering, (c) feeling for the person suffering, (d) tolerating uncomfortable feelings, and (e) motivation to act/acting to alleviate suffering	Yes	Yes	0.90 to 0.94 across all samples
5	Organizational Virtuousness	Rego et al. (2011)	Yes	No, it captured compassion on the basis of stories and compassionate acts in workplace	No	Single factor	No	CFA	0.77
6	Near Scale	Simpson and Farr-Wharton (2017))	Yes	Yes, Near framework of organizational compassion	No	4 factors: Noticing, Empathizing, Assessing, Responding	Yes	Yes	No
7	Team Compassion	Wee and Fehr (2021)	Yes	No, it captured Team compassion	No	Single factor	Yes	Yes	0.94
8	Psychological Compassion Climate	Nolan et al. (2022)	Yes	No, it focused on compassion climate	No	Single factor	Yes	Yes	0.86

As demonstrated in Table 1, there are certain constructs which have been used in the management literature to measure compassion, however, they either lacked a robust psychometric testing or missed out on measuring the key components of workplace compassion which are noticing, empathizing, sensemaking, and acting. One of the first constructs which measured compassion is self-compassion scale (Neff, 2003), the measure focuses on compassion toward oneself and not others (Kotera and Van Gordon, 2021), which is completely different from workplace compassion which is an other-focused construct. Later, experienced compassion scale (Lilius et al., 2008) was introduced in the management literature, however, the focus of the scale was the experience (receiving) of compassion from others instead of giving compassion to others (which is the focus of our study). The organizational virtuous scale (Rego et al., 2011) measured compassion as one of the facets in it, however, this measure focuses on experiencing and witnessing compassion in organization and not on one’s display of compassion toward other individuals in the workplace. Other scales on general compassion such as loving-kindness compassion scale (Cho et al., 2018), compassion scale (Pommier et al., 2020), Sussex-Oxford compassion scale (Gu et al., 2020) did not include workplace-specific factors in their measure. The scales introduced in the management literature such as Near scale (Simpson and Farr-Wharton, 2017), Team compassion scale (Wee and Fehr, 2021), and psychological compassion climate scale (Nolan et al., 2022) either lacked robust psychometric testing or focused on meso or macro level constructs such as team or organizational climate.

Thus, there is a need to develop a reliable and valid measure of workplace compassion that satisfies both conceptual requirements and psychometric testing. We have followed the four-factor conceptualization of organizational compassion which includes noticing, empathizing, sensemaking, and acting (Atkins and Parker, 2012; Dutton et al., 2014). This conceptualization is widely used in the management literature (Dutton et al., 2014; Mascaro et al., 2020; Schabram and Heng, 2021; Wee and Fehr, 2021). Based on this conceptualization, we define workplace compassion as “An individual’s ability to notice, empathize, assess, and act toward the suffering of others with the motivation to alleviate it, in the workplace setting.” These four factors are independently defined as: noticing involves attention to others’ emotions and identifying cues that give awareness of their suffering in the workplace (Kanov et al., 2004; Dutton et al., 2014). Noticing can be through cognitive recognition or by sensing an unconscious physical or emotional reaction to other’s pain in the workplace (Kanov et al., 2004). The suffering of the target can be due to personal or professional difficulties; however, it is noticed in the workplace. Empathizing includes having an empathetic reaction toward others’ suffering and feeling concerns for them. Through empathizing, an individual connects with other person’s suffering which further motivates a desire to alleviate it (Dutton et al., 2014). Sensemaking is the interpretative process where the individual evaluates the sufferer’s conditions on the basis of their deservingness for help, individual’s self-relevance to the sufferer and their condition, and individual’s self-efficacy to act (Atkins and Parker, 2012; Rynes et al., 2012). During sensemaking, an individual assesses their personal values, goal, and future outcomes of acting toward alleviating the suffering of the other party. Acting involves any act or response to alleviate the suffering of the other party (Kanov et al., 2004). It can range from listening to gathering or sharing resources (financial, emotional) to help the sufferer (Dutton et al., 2014). These four processes are part of compassion and are displayed in the workplace setting which is surrounded by job, team and organization level factors. The workplace compassion can be targeted at a co-worker, who as per this article can be a supervisor, subordinate, team member, or any other person who works in the same workplace/organization.

## 3. Materials and methods

The study falls under the positivist paradigm as it considers a single reality that is external and independent which can be measured in an objective manner through proper scientific methods (Zeithaml et al., 2020). Under positivist methodology, one can use survey methods or experiments for quantitative data analysis (Zeithaml et al., 2020). Here, the reality can be captured through scientific methods which are tested in the form of hypothesis. To capture these constructs, data can be collected *via* Likert type scales (Brand, 2009). Our scale follows a multidimensional conceptualization of compassion that emanates from existing literature and theory. The process of scale development and validation follows quantitative method (Rahi, 2017; Tripathi et al., 2022), which spreads over four studies. These four studies are designed based on the best practices and recommendations in psychology and management literature for development and validation of a scale (Hinkin, 1995; MacKenzie et al., 2011; DeVellis and Thorpe, 2021). Study 1 includes item generation based on the existing literature on workplace compassion and by referring to the existing measures on general compassion. Based on the recommendations for content validity (Colquitt et al., 2019), we also checked the content validity of the items generated within the four subconstructs of compassion, i.e., noticing, empathizing, sensemaking, and acting. Study 2 highlights the data purification stage where items that successfully loaded on their respective dimensions were kept while other items which cross-loaded or had lower loading values were removed. Study 3 is conducted to confirm the factor structure of the four-factor model, to assess its model fit. The last study is conducted to test the nomological validity of the measure by checking its correlation with other theoretically-related constructs and other types of compassion. For study 1, we approached the participants based on their qualification; people with advanced degrees in psychology or human-behavior related courses were asked to participate in study 1. For study 2, 3, and 4, we collected data of English speaking, employed population from Prolific. This is consistent with prior studies on scale development (Billard, 2018; Gorbatov et al., 2021; Zhang, 2021). The data collection is done *via* online survey forms which were administered through Qualtrics (Chen et al., 2022). The level of rejecting alpha is (*p* ≤ 0.05).

## 4. Study 1: Item generation and content validation of the workplace compassion scale

The current conceptualization of organizational compassion focuses on a four-factor structure of noticing, empathizing, sensemaking, and acting. The literature on compassion focuses on these four sub-factors as processes of compassionate acting in the workplace, which need not be sequential (Dutton et al., 2014). For the development and validation of the workplace compassion scale, we have followed the same conceptualization of a four-factor structure to measure the compassionate responding in the workplace. As the conceptualization of organizational compassion is unique and has its own literature in management, we have not introduced the sub-factors from other measures on compassion such as Loving-kindness compassion (Cho et al., 2018) and self-compassion (Pommier et al., 2020). The following section deals with the scale development process of the workplace compassion scale (DeVellis and Thorpe, 2021).

### 4.1. Item generation

Items are primarily generated with a deductive approach where the authors have regularly referred to the conceptualization of organizational compassion and its sub-constructs. Additionally, items from the existing measures on compassion, such as the loving-kindness compassion scale (Cho et al., 2018), Sussex-Oxford compassion scale (Gu et al., 2020), compassion scale (Pommier et al., 2020), and other published and unpublished works are also consulted to generate the pool of items. An initial pool of 47 items is generated in the first step (Noticing = 13, Empathizing = 11, Sensemaking = 13, Acting = 10). To ensure the face validity of the initial pool of items, the items were evaluated by one professor and three PhD students who are familiar with the topic of research. They suggested a few language changes in the items which were incorporated in the initial pool.

### 4.2. Content validity

Content validity is an important step as it helps in gaging whether the items generated to measure the construct adequately reflect the content associated with it (Colquitt et al., 2019; Clark et al., 2020). As pointed out in the literature review section, most of the scales measuring compassion (including self and other-oriented compassion) have not given adequate attention to the content validation step. For content validation, we followed the procedure recommended by Anderson and Gerbing (1991), also called Q-methodology or Q-sorting, which incorporates the qualitative assessment of the scale items (Colquitt et al., 2019). This technique is used to evaluate the definitional correspondence (the degree to which the scale’s item align with the definition of the focal construct) and definitional distinctness (the degree to which the scale’s item align with the focal construct in comparison to other constructs) of the item’s generated for the workplace compassion scale (Colquitt et al., 2019). Here, all the items were randomly mixed and presented to a set of raters (PhD students), who were asked to associate these items to their respective construct definitions as per the description given to them.

#### 4.2.1. Participants and procedure

The sample of this study consisted of 10 full-time PhD students, 8 of which belonged to the field of organizational behavior and human resource management, while the remaining two students were from the marketing area. The samples have been reached out through purposive sampling, since we needed participants with knowledge on psychology and human behavior for assessment of the items and their alignment with the sub-constructs. Fifty percent of the participants were female, and the other 50 were male participants. The sample of this study is primarily used for the purpose of content validation with the help of a Q-sort task as recommended by previous research scholars (Anderson and Gerbing, 1991; Colquitt et al., 2019).

#### 4.2.2. Results

The responses collected from the participants were analyzed with Q-sorting technique. Here, we calculated substantive agreement (Psa) and substantive-validity coefficient (Csv) for each item against their proposed definitions (Colquitt et al., 2019). The Psa reflected the agreement of the proportion of the sample for an item that fits its intended construct, items with a Psa less than 0.75 are removed from the pool. The Csv reflected how the participants had assigned a particular item to its intended construct in comparison to other constructs. Items with a Csv less than 0.55 are removed from the pool (Colquitt et al., 2019; Clark et al., 2020). A total of 11 items got eliminated from this process, giving us a final pool of 36 items for EFA.

## 5. Study 2: Item purification

The second study was conducted for confirmatory factor analysis of the 36-item workplace compassion scale. The study served two purposes: (1) to develop the initial factor structure of WCS scale, (2) to refine scale by removing items which cross-loaded or had low loading values.

### 5.1. Data collection

The sample for this study consisted of 413 individuals; however, seven responses were removed from the sample as they failed attention checks. The data for this study is collected *via* Prolific. Finally, the sample consisted of 406 individuals. The participant’s age in the sample varied from 18 to 60 years, while the average age was 30.91 years (SD = 7.3). Out of these 406 participants, 198 (48.8%) were male, 207 (51%) were females, and the remaining 1 participant identified as non-binary/third gender. Participants were majorly Caucasian and African American (Caucasian/White = 44.3%, Black/African American = 44.3% and others 12.4%), with 187 (46.1%) of them having bachelor’s degrees and working at an average of 40.38 h per week.

### 5.2. Results

#### 5.2.1. Exploratory factor analysis

The responses received from the participants are used for conducting exploratory factor analysis in IBM SPSS 20. Here, the participants filled an online survey form containing 36 items of the workplace compassion scale along with their demographic details. We used a seven-point Likert type scale to measure workplace compassion which is anchored from almost never true to almost always true. Items that had an item to total correlation less than 0.5 were removed from the analysis (Kim and Stoel, 2004). Additionally, items that had a correlation of less than 0.3 with any other item of the WCS scale were also removed from the analysis (Callaghan et al., 2015). This is in line with previous research on scale development (Gielnik et al., 2015). This process resulted in the elimination of 20 items from the pool, leaving 16 items for further analysis.

The exploratory factor analysis was performed with principal axis factoring and promax rotation on the pool of 16 items received from the previous step. The promax rotation is chosen as the sub-dimension are correlated with each other, this is in line with previous studies on scale development (Luchs et al., 2021; Luse and Burkman, 2022; Schaarschmidt et al., 2022). The appropriation of conducting EFA was confirmed with Kaiser-Meyer-Olkin Measure of Sampling Adequacy (0.932) and Bartlett’s Test of Sphericity [*χ*^2^(4050.65) = 120, *p* < 0.001; Luse and Burkman, 2022]. As per the recommendations by Wolfinbarger and Gilly (2003), we only retained the items which: (1) demonstrated a loading of 0.5 or higher, (2) did not have a cross-loading of 0.5 or higher. This resulted in the elimination of 4 items from the proposed pool, giving us a set of 12 items for further analysis. These 12 items loaded in their respective factors with a loading value of more than 0.5 with no cross-loading. The four-factor solution of EFA explained a total variance of 68.64%. The results of the exploratory factor analysis including factor matrix and item loading are displayed in Table 2.

**Table 2:** Rotated factors and loadings of retained items from study 2 (exploratory factor analysis with promax rotation) (*N* = 406).

Items	Workplace compassion			
	Noticing	Empathizing	Sensemaking	Acting
I notice my co-worker’s feelings of distress or pain without them having to tell me.	0.834			
I notice the feeling of discomfort experienced by people in my workplace.	0.590			
I recognize people’s feelings of distress without them having to tell me in my workplace.	0.996			
I can feel the distress as my own when I see someone in my workplace experiencing it.		0.583		
I experience pain from people’s distress in my workplace.		0.926		
I feel the pain experienced by people in my workplace.		0.816		
I try to assess the outcomes of my actions toward the person’s distress in my workplace.			0.0.637	
I try to assess the prior circumstances leading to the person’s suffering in my workplace.			0.978	
I try to understand the context of the person’s distress in my workplace.			0.530	
I initiate offering help to people in my workplace, when I hear the story of their pain.				0.769
When I see someone in distress in my workplace, I try to act as quickly as possible.				0.814
When I see someone in my workplace in pain, I try to help them.				0.827

#### 5.2.2. Preliminary internal consistency

The Cronbach’s alpha for the overall workplace compassion scale with 12 items is 0.915 for the sample of this study. The Cronbach’s alpha for each dimension of workplace compassion is also found to be suitable: noticing 0.875, empathizing 0.849, sensemaking 0.823, acting 0.873.

## 6. Study 3: Factor structure

The third study was conducted for the purpose of item purification of the 12-item workplace compassion scale. The study served two purposes: (1) To assess the model fit of the proposed factor structure, (2) To assess reliability and validity of WCS.

### 6.1. Data collection

The sample for this study consisted of 280 individuals; however, 4 participants out of this were removed because they failed attention checks. The data for this study is collected *via* Prolific. The final no. of participants in the study was 276, the average age of the participants was 30.51, with 49.3% of them being male and the other 47.8% being females. Out of 276 participants, 61.6% were Caucasians, 36.6% of the participants have bachelor’s degrees and were working at an average of 32.46 h per week. The sample of this study was used for confirmatory factor analysis.

### 6.2. Results

#### 6.2.1. Confirmatory factor analysis

The data from the respondents is used for confirmatory factor analysis, which is done with the help of IBM SPSS AMOS 23. For CFA, a four-factor model of workplace compassion is created keeping in mind the conceptualization and its psychometric multidimensionality, which includes its unique subconstructs, i.e., noticing, empathizing, sensemaking, and acting. The initial four-factor model was tested with 12 items retained from the EFA and multiple fit indices were assessed for the same. The fit indices included chi-square/degree of freedom, Goodness of Fit (GFI), Adjusted Goodness of Fit (AGFI), comparative fit index (CFI), Root Mean Square Residual (SRMR), Root-Mean-Square Error of Approximation (RMSEA), and Tucker–Lewis Fit Index (TLI) (Hu and Bentler, 1999). The 12-item, four-factor model showed acceptable fit indices: *χ*^2^/df = 112.057/59, GFI = 0.941, AGFI = 0.909, CFI = 0.982, SRMR = 0.051, RMSEA = 0.057, TLI = 0.976. The item loading for the CFA is presented in Table 3.

**Table 3:** Factors Loadings of confirmatory factor analysis for study 3 (*N* = 276).

Items	Factor loadings
Noticing	
I notice my co-worker’s feelings of distress or pain without them having to tell me.	0.848
I notice the feeling of discomfort experienced by people in my workplace.	0.769
I recognize people’s feelings of distress without them having to tell me in my workplace.	0.952
Empathizing	
I can feel the distress as my own when I see someone in my workplace experiencing it.	0.758
I experience pain from people’s distress in my workplace.	0.910
I feel the pain experienced by people in my workplace.	0.894
Sensemaking	
I try to assess the outcomes of my actions toward the person’s distress in my workplace.	0.833
I try to assess the prior circumstances leading to the person’s suffering in my workplace.	0.853
I try to understand the context of the person’s distress in my workplace.	0.824
Acting	
I initiate offering help to people in my workplace, when I hear the story of their pain.	0.875
When I see someone in distress in my workplace, I try to act as quickly as possible.	0.865
When I see someone in my workplace in pain, I try to help them.	0.882

#### 6.2.2. Convergent/discriminant validity findings

The convergent validity can be examined in two ways. First, the item level convergent validity of the scale can be assessed by checking whether the item correctly load onto their hypothesized (conceptualized) factor at a value above the threshold of 0.5. As presented in Table 3, the items loading value of each item is above 0.75, which satisfies the first condition for convergent validity. Second, the factor-level convergent validity of the scale is required to be assessed to ensure each factor has a unique contribution to the scale. For this, we checked whether the composite reliability of each factor is higher than its Average variance extracted (AVE), which should be higher than 0.5 for each factor. Table 4 represents the composite reliability and AVE for each factor.

**Table 4:** Convergent and discriminant validity analysis from study 2 (*N* = 276).

Factor	CR	AVE	MSV	Noticing	Empathizing	Sensemaking	Acting
Noticing	0.894	0.739	0.364	0.86			
Empathizing	0.892	0.734	0.556	0.538***	0.857		
Sensemaking	0.875	0.7	0.677	0.604***	0.745***	0.836	
Acting	0.907	0.764	0.677	0.513***	0.695***	0.823***	0.874

CR = Composite Reliability; AVE = Average Variance Extracted; MSV = Maximum Shared Variance; Square root of AVE on the diagonal; * *p* < 0.050; ** *p* < 0.010; *** *p* < 0.001.

We further assessed the discriminant validity by checking whether the square root of the AVE for a particular factor is higher than its correlation with any other factor in the scale. Additionally, the maximum shared variance was observed to be less than the average variance extracted. Table 4 represents the results of the discriminant validity test, and it can be seen that the proposed condition is met for all four factors. To further assess the discriminant validity, we compared the model fit measures of the proposed four-factor correlated model with other three-factor, two-factor, and one-factor models. As shown in Table 5, the four-factor correlated model demonstrated the best model fit measures compared to any other alternative model.

**Table 5:** Model fit measures-confirmatory factor analysis for study 2 (*N* = 276).

Model	*χ*^2^/df	df	IFI	CFI	SRMR	RMSEA	AIC	TLI	GFI	AGFI
Four-factor correlated model (WCS)	2.014	48	0.981	0.981	0.051	0.061	156.686	0.973	0.945	0.911
Three-factor models										
Noticing-empathizing combined	8.515	51	0.848	0.847	0.083	0.165	488.254	0.802	0.778	0.660
Noticing-sensemaking combined	7.786	51	0.863	0.862	0.089	0.157	451.099	0.821	0.795	0.687
Noticing-Acting combined	9.340	51	0.831	0.830	0.107	0.174	530.325	0.780	0.763	0.638
Empathizing-sensemaking combined	4.797	51	0.923	0.923	0.52	0.118	298.657	0.900	0.862	0.790
Empathizing-acting combined	5.985	51	0.899	0.898	0.063	0.135	359.217	0.869	0.826	0.734
Sensemaking-acting combined	4.082	51	0.938	0.937	0.064	0.106	262.184	0.919	0.869	0.799
Two-factor models										
Noticing-empathizing-sensemaking combined	10.208	53	0.806	0.805	0.092	0.183	591.008	0.757	0.750	0.631
Noticing-empathizing-Acting combined	11.955	53	0.769	0.768	0.101	0.200	683.592	0.711	0.708	0.571
Noticing-sensemaking-acting combined	10.216	53	0.806	0.805	0.103	0.183	691.443	0.757	0.738	0.615
Empathizing-sensemaking-acting combined	7.169	53	0.873	0.873	0.067	0.148	421.936	0.841	0.801	0.707
One-factor model	12.768	54	0.747	0.746	0.104	0.207	737.497	0.690	0.696	0.561

*χ*^2^ = Chi-square test of exact fit; df = Degrees of freedom; IFI = Incremental fit index; CFI = comparative fit index; SRMR = Standardized root mean square residual; RMSEA = Root mean square error of approximation; AIC = Akaike information criterion; TLI = Tucker–Lewis’s index; GFI = Goodness of fit; AGFI = Adjusted goodness of Fit.

#### 6.2.3. Internal consistency

The Cronbach’s alpha for the overall workplace compassion scale with 12 items is 0.93 for the sample of this study. The Cronbach’s alpha for each dimension of workplace compassion is also found to be suitable: noticing 0.887, empathizing 0.883, sensemaking 0.874, acting 0.905.

## 7. Study 4: Nomological network

This study focused on establishing the nomological validity of the proposed measure of workplace compassion. Nomological validity is important as it helps in confirming that the construct of interest fits in the existing theoretical network (Böttger et al., 2017). The aim of nomological network is not to build theory but to test existing relationships of the construct (Böttger et al., 2017). This is done through a time-lagged research design with a gap of 3 days between the proposed constructs. Three days delay is ideal for temporally separated studies measuring psychological constructs such as ability, attitude, and others. It is significant enough to allow the psychological phenomena to unravel. Three days gap is neither too long (where other factors may influence the results), nor too short (where the effect has not fully manifested). It also provides enough no. of buffer days in between the measured constructs and takes care of repetition on the same day effect like weekly separated studies (because same week-day can influence the results). Here, one of the antecedents of organizational compassion, perceived organizational support is captured at T1, workplace compassion (WC) is measured at T2 (T1 + 3 days), and work engagement which is a possible outcome of WC is measured at T3 (T2 + 3 days).

The antecedent of workplace compassion perceived organizational support (POS) is drawn from the literature on organizational compassion (Atkins and Parker, 2012; Dutton et al., 2014; Simpson et al., 2015). The employee’s perception of organizational support is grounded on their evaluation of whether the organization actually value their contribution (Eisenberger et al., 1986), which is reflected in the form of the organization’s concern toward individual well-being at workplace (Rhoades and Eisenberger, 2002). Existing research indicates that POS invokes felt obligation in the employee, which is reciprocated by the employee in the form of activities that support their fellow workers and organization in achieving its goal. Based on this, we hypothesize:

*H1*: Perceived organizational support is positively associated with workplace compassion.

The existing research on compassion indicates through affective events theory that compassionate acts embeds an individual in the workplace and also results in them experiencing positive emotions (Lilius et al., 2008). Individuals who experience positive emotions tend to show higher engagement at work because of the said benefits. Existing research on compassion already confirms a positive association between experiencing compassion and work engagement (Buonomo et al., 2021). Based on this, we hypothesize:

*H2*: Workplace compassion is positively associated with workplace engagement.

### 7.1. Data collection

The sample of the study consists of 255 participants. The data for this study is collected *via* Prolific. We started with 307 participants for T1, where the participants indicated their response on the perceived organizational support and demographics. At T2, for workplace compassion, we received responses from 282 individuals. Finally, at T3, responses were collected for work engagement and a total of 260 individuals participated in this study. We did check for non-response bias between the data collected at T1, T2, and T3; we did not observe any significant differences between those who completed all three surveys and those who did not. After removing the participants who failed the attention check and the outliers in the data, the final sample consisted of 255 participants. The participant’s ages varied from 21 to 62 while the mean age was 36.53 years. Out of 255 participants, 129 (50.5%) were male, 121 (47.5%) were female, and 5 (2%) identified as other. Around 72% of participants had a bachelor’s degree or higher in education and 58.4% of the participants worked in the service industry. The average experience of participants in their present organization was 65.5 months, while the participants worked for an average of 41.5 hours weekly.

### 7.2. Measures

The following measures are used in this study. The Cronbach’s alpha of these scale are presented in Table 6.

**Table 6:** Means, standard deviations, correlations, and reliabilities for nomological validity (*N* = 255).

Variables	Mean	Std. Deviation	1	2	3	4	5	6	7	8	9	10	11	12	13	14
1. Noticing	4.928	1.203	(0.929)													
2. Empathizing	4.052	1.368	0.546**	(0.938)												
3. Sensemaking	4.595	1.306	0.511**	0.637**	(0.899)											
4. Acting	4.912	1.244	0.528**	0.536**	0.650**	(0.920)										
5. WCS	4.622	1.054	0.777**	0.836**	0.854**	0.821**	(0.93)									
6. POS	4.661	1.621	0.023	0.083	0.096	0.184**	0.118	(0.96)								
7. WE	4.490	1.426	0.177**	0.152*	0.208**	0.328**	0.261**	0.526**	(0.883)							
8. SC	4.280	1.264	0.086	0.059	0.138*	0.166*	0.135*	0.385**	0.430**	(0.867)						
9. EXPC	4.676	1.335	0.191**	0.217**	0.276**	0.312**	0.302**	0.590**	0.529**	0.271**	(0.853)					
10. Age	36.530	9.979	0.015	−0.006	−0.04	0.090	0.016	0.157*	0.148*	0.135*	0.067					
11. Gender	1.510	0.539	0.181**	0.122	0.043	0.065	0.124*	−0.106	−0.105	−0.170**	0.021	−0.055				
12. Education	4.740	1.405	−0.035	−0.038	−0.016	0.048	−0.013	−0.037	0.061	0.05	0.026	0.091	−0.029			
13. Industry	2.140	0.630	0.015	0.01	0.059	0.041	0.038	0.021	0.06	−0.011	0.084	−0.016	0.064	0.109		
14. WRK-P	65.550	66.774	0.038	0.045	0.025	0.058	0.050	−0.011	0.078	0.031	−0.007	0.427**	−0.095	0.037	0.098	
15. WRK-W	41.490	6.963	0.181**	0.138*	0.124*	0.180**	0.188**	−0.085	0.047	−0.023	−0.074	0.197**	−0.015	0.132*	0.09	0.141*

WCS = workplace compassion scale; POS = Perceived organizational support; WE = Work engagement; SC = Self-compassion; EXPC = experienced compassion; WRK-P = Tenure in present organization; WRK-W = Weekly working hours; Cronbach’s alpha in the parenthesis; *p < 0.05; **p < 0.01.

Workplace compassion: workplace compassion is measured with the 12-item scale developed in the previous study. The scale consisted of four sub-dimensions: noticing, empathizing, sensemaking, and acting. Participants were asked to report the degree to which they relate to other people in the workplace (1 = Almost never true, 7 = Almost always true). A sample item is I initiate offering help to people in my workplace, when I hear the story of their pain.

Perceived organizational support: The perceived organizational support is captured with an 8-item scale of POS (Eisenberger et al., 1986). Participants indicated their degree of agreement / disagreement on the scale items (1 = Strongly disagree, 7 = Strongly agree). A sample item is “The organization values my contribution to its well-being.”

Work engagement: Work engagement is measured with a 3-item short work engagement scale (Schaufeli et al., 2017). The scale is anchored from Never (1) to Always (7). A sample item is “At my work, I feel bursting with energy.”

Self-compassion: To measure self-compassion, we used a 6-item short state self-compassion scale (Neff et al., 2021). The scale is anchored from not at all true of me (1) to very true of me (7). A sample item is “I’m giving myself the caring and tenderness I need.”

Experienced compassion: The experienced compassion at work is measured with a 3-item scale (Lilius et al., 2008). The scale is anchored from never (1) to nearly all the time (7). A sample item is “Indicate how frequently you have experienced compassion: on the job.”

Ten-item personality scale: We used the 10 item personality scale to measure the big five personality traits (Gosling et al., 2003).

### 7.3. Results

Mean, standard deviations, and inter-construct correlation between the constructs of the study, sub-constructs of compassion, and control variables are presented in Table 6. We used the following groupings to evaluate the factor structure of our scale: first, with perceived organizational support (antecedent) and work engagement (outcome) to confirm the nomological network of workplace compassion scale. Second, with experienced compassion and self-compassion to confirm the discriminant validity from related constructs of the workplace compassion scale. We build the factor structure and tested it with confirmatory factor analysis to assess the model fit indices along with convergent and discriminant validity.

The model fit indices for the nomological network displayed satisfactory model fit: χ2/df = 405.221/215, CFI = 0.966, SRMR = 0.042, RMSEA = 0.059, TLI = 0.960. Table 7 presents the convergent and discriminant validity for the nomological network, and no validity concern has been observed in the results. To evaluate the nomological network, we used Process Macro v3.5 (Hayes, 2017) to create valid coefficients that determine the association between our constructs (Thompson et al., 2020). After controlling for age, gender, education, industry, tenure in the present organization, and the number of work hours in a week, we found a positive association between POS and workplace compassion (*β* = 0.1614, *p* < 0.05), which supported the hypothesis 1. Similarly, we also observed a positive association between workplace compassion and work engagement (*β* = 0.2048, *p* < 0.001), which supported hypothesis 2. The results of the analysis are presented in Table 8.

**Table 7:** Convergent and discriminant validity analysis for nomological network (*N* = 255).

Factors	CR	AVE	MSV	POS	Acting	Empathizing	Noticing	Work engagement	Sensemaking
POS	0.961	0.754	0.330	0.869					
Acting	0.921	0.796	0.494	0.195**	0.892				
Empathizing	0.939	0.837	0.453	0.091	0.554***	0.915			
Noticing	0.931	0.817	0.324	0.023	0.569***	0.567***	0.904		
Work engagement	0.887	0.726	0.330	0.574***	0.370***	0.133*	0.177**	0.852	
Sensemaking	0.904	0.76	0.494	0.108	0.703***	0.673***	0.546***	0.214**	0.871

POS = Perceived organizational support; CR = Composite Reliability; AVE = Average Variance Extracted; MSV = Maximum Shared Variance; Square root of AVE on the diagonal; * *p* < 0.05; ** *p* < 0.01; *** *p* < 0.001.

**Table 8:** Results of the regression analysis for nomological network (*N* = 255).

	Workplace compassion	Work engagement
Control variables		
Age	−0.0685	0.0357
Gender	0.1460*	−0.0723
Education	−0.0268	0.0686
Industry	0.0013	0.0332
WRK-P	0.0663	0.0413
WRK-W	0.2113**	0.0241
Independent variables		
Perceived organizational support (POS)	0.1614*	0.4932***
Workplace compassion		0.2048***
Adjusted R square	0.0793**	0.3365***
*F*	*3.0403*	*15.5967*

*p < 0.05, **p < 0.01, ***p < 0.001.

Additionally, the model for testing discriminant validity with other related constructs of compassion such as self-compassion and experienced compassion displayed satisfactory model fit indices: *χ*^2^/df = 327.359/174, CFI = 0.961, SRMR = 0.057, RMSEA = 0.059, TLI = 0.953. Table 9 presents the convergent and discriminant validity for the said factor structure, and no validity concerns have been observed in the results. We further used a more stringent heterotrait-monotrait (HTMT) method of discriminant validity testing; here, we examined the disattenuated correlations between the constructs (Walsh et al., 2019; Grace et al., 2020). An HTMT value of less than 0.85 indicates discriminant validity between the said constructs (Walsh et al., 2019). Table 10 presents the results of HTMT analysis, and it can be seen that no discriminant validity violations are observed in the data. We also tested the correlation of self-compassion and experienced compassion with our proposed workplace compassion. The self-compassion yielded a positive correlation with workplace compassion (*r* = 0.135, *p* < 0.05), however, the correlation is not very high (correlation of 0.8 or above (Kline, 2005)) which suggests less overlap between these constructs. The experienced compassion also showed a significant positive correlation with workplace compassion (*r* = 0.302, *p* < 0.001), however, the value was not very high which suggests less overlap between the constructs.

**Table 9:** Convergent and discriminant validity analysis for differentiating other related constructs (*N* = 255).

Factors	CR	AVE	MSV	Acting	Self-compassion	Empathizing	Noticing	Experienced compassion	Sensemaking
Acting	0.921	0.796	0.495	0.892					
Self-compassion	0.871	0.533	0.108	0.175*	0.73				
Empathizing	0.939	0.837	0.454	0.554***	0.065	0.915			
Noticing	0.931	0.817	0.324	0.569***	0.11	0.567***	0.904		
Experienced compassion	0.857	0.668	0.115	0.340***	0.328***	0.241***	0.188**	0.817	
Sensemaking	0.904	0.759	0.495	0.703***	0.147*	0.674***	0.546***	0.311***	0.871

CR = Composite Reliability; AVE = Average Variance Extracted; MSV = Maximum Shared Variance; Square root of AVE on the diagonal; * *p* < 0.05; ** *p* < 0.01; *** *p* < 0.001.

**Table 10:** HTMT assessment of discriminant validity analysis for differentiating other related constructs (*N* = 255).

	Acting	Self-compassion	Empathizing	Noticing	Experienced compassion	Sensemaking
Acting						
Self-compassion	0.188					
Empathizing	0.577	0.074				
Noticing	0.572	0.099	0.585			
Experienced compassion	0.355	0.32	0.245	0.217		
Sensemaking	0.716	0.165	0.692	0.557	0.319	

### 7.4. Personality correlates

In line with previous studies on scale development, we checked the personality correlates of the workplace compassion scale and its dimensions (Clark et al., 2020). We also checked if personality dimensions are associated with workplace compassion, this is done in line with previous literature on compassion and personality traits (Neff et al., 2007; Thurackal et al., 2016; Di Fabio and Saklofske, 2021). We used the 10-item Big five questionnaire to measure the personality traits of the participants (Rammstedt and John, 2007). We used the same sample of 255 participants for this. Table 11 presents the mean, standard deviation, and inter-construct correlation between the big five personality traits (extraversion, agreeableness, openness, conscientiousness, and neuroticism) and workplace compassion and its sub-dimensions. The results of the bivariate correlation indicates that workplace compassion was positively correlated with extraversion (*r* = 0.198, *p* < 0.001), agreeableness (*r* = 0.334, *p* < 0.001), conscientiousness (*r* = 0.169, *p* < 0.001), openness (*r* = 0.228, *p* < 0.001) which is in line with previous literature on compassion (Neff et al., 2007; Thurackal et al., 2016; Di Fabio and Saklofske, 2021). However, we did not observe any significant correlation between workplace compassion and neuroticism.

**Table 11:** Means, standard deviations, and correlations for personality correlates (*N* = 255).

Variables	Mean	Std. Deviation	1	2	3	4	5	6	7	8	9	10
1. Noticing	4.9281	1.20315	1									
2. Empathizing	4.0523	1.36794	0.546**	1								
3. Sensemaking	4.5948	1.30601	0.511**	0.637**	1							
4. Acting	4.9124	1.24466	0.528**	0.536**	0.650**	1						
5. WCS	4.6219	1.05388	0.777**	0.836**	0.854**	0.821**	1					
6. Neuroticism	4.7059	1.51169	−0.085	0.029	0.068	0.098	0.035	1				
7. Conscientiousness	5.6510	1.12669	0.239**	0.061	0.100	0.170**	0.169**	0.275**	1			
8. Extraversion	3.5216	1.67324	0.211**	0.100	0.101	0.252**	0.198**	0.255**	0.115	1		
9. Agreeableness	5.2608	1.25320	0.177**	0.309**	0.237**	0.371**	0.334**	0.438**	0.205**	0.165**	1	
10. Openness	5.1725	1.25633	0.230**	0.157*	0.153*	0.214**	0.228**	0.319**	0.235**	0.274**	0.307**	1

*p < 0.05; **p < 0.01.

Additionally, we also conducted the predictive validity of personality correlates with workplace compassion (Neff et al., 2007; Thurackal et al., 2016). We used hierarchical multiple regression for this. The regression results demonstrated that extraversion (*β* = 0.154, *p* < 0.01), openness (*β* = 0.132, *p* < 0.05), and agreeableness (*β* = 0.336, *p* < 0.001) had significant positive association with workplace compassion. Neuroticism showed a significant negative association with workplace compassion (*β* = −0.246, *p* < 0.001) while conscientiousness had no significant association with workplace compassion.

## 8. Discussion

Compassion has a long history of literature on it, where people from different scholarly backgrounds have emphasized its positive effects on the individual, society, and the overall world (Gilbert, 2020). The literature on compassion emphasizes suffering as an inherent part of the compassionate responding process (Gilbert et al., 2019). Compassion as a phenomenon of interest found its way into the management literature trough the research works on organizational compassion (Frost, 1999). Organizational compassion is becoming a prominent area of research in management as it normalizes the expression of both positive and negative emotions in the workplace and supports mutual thriving along with other positive individual and organizational outcomes (Tsui, 2013; Dutton et al., 2014). This research demonstrates the development and validation of the workplace compassion scale with the help of four studies. We followed the conceptualization proposed by scholars in the literature of organizational compassion to develop the initial sub-dimensions and items of the workplace compassion scale (Atkins and Parker, 2012; Dutton et al., 2014). The four-factor, 12 item scale retained the current conceptualization of organizational compassion that consists of noticing, empathizing, sensemaking, and acting. The model fit indices displayed good acceptability. Additionally, the internal consistency of the whole scale, as well as its sub-constructs, also showed satisfactory levels of acceptance across studies.

Most of the studies on organizational compassion has been carried out with the help of qualitative techniques and scales that needed a formal reliable and valid testing procedure (Lilius et al., 2008; DeCelles and Anteby, 2020; Schabram and Heng, 2021). This lacuna in the literature on organizational compassion has contributed to having a limited number of studies on compassion in the workplace and understanding its antecedents and outcomes (Dutton et al., 2014). Research on organizational compassion has also focused on theory building and development; however, theory testing is still in its growing stage (Simpson et al., 2015)(Atkins and Parker, 2012; Madden et al., 2012; Rynes et al., 2012). A valid and reliable scale on workplace compassion is particularly valuable in this context as it can help gauge the importance of compassion in the workplace and its effects on the compassion-receiver and compassion-giver (Dutton et al., 2014). Additionally, our scale can contribute to understanding cross-cultural differences in compassionate responding at work and the relational nature of compassion in an organizational setting when used with other constructs of compassion such as experienced compassion (Dutton et al., 2014; Gilbert, 2019).

## 9. Limitations and future direction

This research is not free from limitations. First, the data for the study is collected from Prolific and we kept the sample limited to English speaking countries (majorly United States); future researchers can go for comparative cultural testing of the proposed scale as the results might change in Asian countries due to the cultural influence of religion (Buddhism) and collectivism. This can be a threat to the external validity of the scale as compassion can be shaped by socio-cultural and religious factors (Pandey and Singh, 2015a,b; Sinha et al., 2017). Second, we have used one antecedent and one outcome in the nomological validity, future researchers can also look for other constructs and their relationship with workplace compassion. Third, organizational compassion, like other virtuous qualities, is not free from social desirability bias, and thus, for future research, social desirability can be checked while capturing compassion in the workplace.

## 10. Conclusion

Organizational compassion talks about the enablers and disablers of compassion in the workplace as the expression of compassion is affected by different organizational contexts such as role, status, culture, norms, and practices (Dutton et al., 2014). Having a psychometric tool to study workplace compassion helps both the researchers and practitioners understand how the job, team, and organizational factors affect compassionate responding in the work settings (Martin et al., 2015). Practitioners can also use this scale to understand the level of compassionate responding in the workplace in addition to general employee outcomes. To develop the workplace compassion scale, we adopted four different studies that started from item generation to validating the nomological network of the workplace compassion scale. There is a need to make workplaces more compassionate in the current times when we are faced with many unimaginable global challenges (Benevene et al., 2022). Current research shows how focusing on compassion in the workplace can enhance job resources (Buonomo et al., 2021), happiness at work and personal lives (Yao et al., 2021), and increase job outcomes (Wan et al., 2022). A valid and reliable measure can open a plethora of research possibilities to further explore benefits of compassion in the workplace and its impact on individuals’ lives.

## Data availability statement

The raw data supporting the conclusions of this article will be made available by the authors, without undue reservation.

## Ethics statement

Ethical review and approval was not required for the study on human participants in accordance with the local legislation and institutional requirements. The patients/participants provided their written informed consent to participate in this study.

## Author contributions

All authors listed have made a substantial, direct, and intellectual contribution to the work and approved it for publication.

## Conflict of interest

The authors declare that the research was conducted in the absence of any commercial or financial relationships that could be construed as a potential conflict of interest.

## Publisher’s note

All claims expressed in this article are solely those of the authors and do not necessarily represent those of their affiliated organizations, or those of the publisher, the editors and the reviewers. Any product that may be evaluated in this article, or claim that may be made by its manufacturer, is not guaranteed or endorsed by the publisher.

## Appendix A

Workplace compassion scale.

*Below are statements describing how you might relate to other people at work. Please indicate how true the following statements are for you; using the 7-point response scale from almost never true to almost always true.*

The term co-worker here signifies the respondent’s supervisor, subordinate, team member, or any other person who works in the same workplace/organization with the respondent (compassion giver).

**Table:**

Almost never true	Usually not true	Rarely true	Occasionally true	Often true	Usually true	Almost always true
1	2	3	4	5	6	7

1. I experience pain from people’s distress in my workplace. (E2)
2. When I see someone in distress in my workplace, I try to act as quickly as possible. (A2)
3. I recognize people’s feelings of distress without them having to tell me in my workplace. (N3)
4. I can feel the distress as my own when I see someone in my workplace experiencing it. (E1)
5. I notice the feeling of discomfort experienced by people in my workplace. (N2)
6. I try to assess the prior circumstances leading to the person’s suffering in my workplace. (S2)
7. I feel the pain experienced by people in my workplace. (E3)
8. I try to assess the outcomes of my actions toward the person’s distress in my workplace. (S1)
9. I initiate offering help to people in my workplace, when I hear the story of their pain. (A1)
10. I notice my co-worker’s feelings of distress or pain without them having to tell me. (N1)
11. I try to understand the context of the person’s distress in my workplace. (S3)
12. When I see someone in my workplace in pain, I try to help them. (A3)

N = Noticing, E = Empathizing, S = Sensemaking, A = Acting.
